# Artificial escape from XCI by DNA methylation editing of the *CDKL5* gene

**DOI:** 10.1093/nar/gkz1214

**Published:** 2020-01-11

**Authors:** Julian A N M Halmai, Peter Deng, Casiana E Gonzalez, Nicole B Coggins, David Cameron, Jasmine L Carter, Fiona K B Buchanan, Jennifer J Waldo, Samantha R Lock, Johnathon D Anderson, Henriette O’Geen, David J Segal, Jan Nolta, Kyle D Fink

**Affiliations:** 1 Department of Neurology, University of California Davis School of Medicine, Sacramento, CA 95817, USA; 2 Stem Cell Program and Gene Therapy Center, University of California, Davis, Sacramento, CA, USA; 3 Genome Center and Department of Biochemistry and Molecular Medicine, University of California, Davis, CA 95616, USA; 4 Department of Otolaryngology, University of California, Davis, Davis, CA, USA

## Abstract

A significant number of X-linked genes escape from X chromosome inactivation and are associated with a distinct epigenetic signature. One epigenetic modification that strongly correlates with X-escape is reduced DNA methylation in promoter regions. Here, we created an artificial escape by editing DNA methylation on the promoter of *CDKL5*, a gene causative for an infantile epilepsy, from the silenced X-chromosomal allele in human neuronal-like cells. We identify that a fusion of the catalytic domain of TET1 to dCas9 targeted to the *CDKL5* promoter using three guide RNAs causes significant reactivation of the inactive allele in combination with removal of methyl groups from CpG dinucleotides. Strikingly, we demonstrate that co-expression of TET1 and a VP64 transactivator have a synergistic effect on the reactivation of the inactive allele to levels >60% of the active allele. We further used a multi-omics assessment to determine potential off-targets on the transcriptome and methylome. We find that synergistic delivery of dCas9 effectors is highly selective for the target site. Our findings further elucidate a causal role for reduced DNA methylation associated with escape from X chromosome inactivation. Understanding the epigenetics associated with escape from X chromosome inactivation has potential for those suffering from X-linked disorders.

## INTRODUCTION

Epigenetics is the study of mitotically and/or meiotically stable but reversible modifications to nucleotides or higher order chromatin structure that can alter expression patterns of genes in the absence of changes to the underlying DNA sequence (1). These modifications occur on multiple levels, such as 5-methyl-cytosine (5-meC) DNA methylation, post-translational modifications of histones bound by protein domains that serve as epigenetic writers, readers and erasers and noncoding RNAs that assist in the recruitment of chromatin modifying proteins to DNA (2). These epigenetic layers dynamically dictate the three-dimensional organization of the genome within the nuclear ultrastructure and orchestrate local accessibility for the eukaryotic transcriptional machinery (3). Because of this, epigenetic signatures play a crucial role in dictating cellular identity during development and throughout life in response to the environment (1), correlate with aging (4) and are linked to disease (5). The process of X-chromosome inactivation (XCI) epigenetically regulates the amount of transcriptionally active X-chromatin in somatic tissue as a dosage compensation mechanism to ensure equal expression levels of X-linked genes in males and females (6). In female somatic cells, one X chromosome randomly becomes inactive and is cytologically manifested during interphase as a perinuclear heterochromatic Barr body, which is then clonally maintained through mitosis (7,8). This mechanism is mediated by the long noncoding RNA X-inactive specific transcript (XIST) expressed from the inactive X chromosome in *cis* (9), which serves as a guiding factor to tether Polycomb proteins for gene silencing to target sites on the X-chromatin (10). XIST induces the formation of repressive heterochromatin through histone deacetylation (11), DNA methylation of CpG-island (CGI) promoters (12), di- and trimethylation of histone 3 at lysine 9 (H3K9me2/3) (13), the deposition and spreading of H3K27me3 across the inactive X-chromatin (14) and the H2A histone variant macroH2A (15).

Interestingly, gene expression data suggests there is an estimated 15–30% of human X-linked genes that escape XCI (16) at an arbitrary transcriptional threshold of 10% of the active allele (17). The level of escape from XCI is variable between genes and individuals (16), demonstrates tissue heterogeneity (18) and increases with age (19). Strikingly, X-escapees have a distinct epigenetic signature from genes that are subject to XCI, including enrichment of active and depletion of repressive histone marks, and generally reduced levels of DNA methylation near regulatory elements (17). In particular, the degree of CGI promoter 5-meC DNA methylation has been demonstrated to be highly correlative with XCI (12,20). In line with the idea that DNA methylation forms an epigenetic barrier on the inactive X chromosome, the most potent X-reactivation to date has been achieved by treatment with 5-azacytidine, a global DNA hypomethylating agent in combination with X-wide genetic ablation of *XIST* (21). In addition, pharmacological and genetic screens aiming to identify *trans*-acting factors promoting XCI have identified the maintenance DNA methyltransferase DNMT1 as a key player in XCI (22,23). However, previous studies aiming to elucidate the mechanism of XCI-escape, such as the aforementioned small molecule approaches, utilized untargeted approaches. While these studies have provided a significant foundation of knowledge, in particular demonstrating the importance of DNA methylation in our understanding of X-reactivation, the global side-effects of these types of approaches limit the study of specific gene reactivation. In order to further our understanding of XCI and reactivation of X-linked genes, targeted approaches that result in specific gene reactivation are required.

Until recently, the lack of targeted approaches by which we can modify epigenetics has limited the ways in which we can understand mechanisms of XCI. With the availability of the RNA-guided clustered regularly interspaced palindromic repeats (CRISPR) system, catalytically inactive dCas9 fused to epigenetic effector domains has become the method of choice for targeted rewriting of the epigenome to further elucidate the causality between epigenetic marks and gene expression (24,25). Recently, dCas9 fusions with the catalytic domain of ten-eleven translocation dioxygenase 1 (TET1) have gained prominence as a candidate to precisely demethylate gene promoters or enhancers for multiple gene targets (26–29). Targeted DNA demethylation of genes on the X chromosome would allow for a directed assessment of the causal role between DNA methylation and gene expression on the inactive X chromosome. Furthermore, the presence of coding SNPs that exist in clonally-derived female cell lines provides an allele-specific model to study escape from XCI induced by targeted epigenetic remodelling.

The neurodevelopmental disorder *CDKL5* deficiency is caused by *de novo* mutations in the *CDKL5* gene on the X chromosome (30). Due to random XCI, females affected by the disorder form a mosaic of tissue with cells expressing either the mutant or wild type allele (31). A potential therapeutic approach might be to activate the silenced wild type *CDKL5* allele in cells expressing the loss-of-function mutant allele. Here, we sought to synthetically induce escape of *CDKL5* from the inactive X chromosome in the neuronal-like cell line SH-SY5Y. DNA methylation editing of the *CDKL5* promoter using a dCas9-TET1 fusion protein for targeted DNA demethylation was found to result in a significant increase in allele-specific expression of the inactive allele and is correlated with a significant reduction in methylated CpG dinucleotides in the CGI core promoter. We also demonstrate that TET1 has a synergistic effect with the transcriptional activator VP64, thereby further increasing transcript levels from the inactive allele. As such, our study demonstrates that loss of DNA methylation is crucial for inducing escape from the inactive X chromosome. Finally, we confirmed with whole-transcriptomic and genome-wide methylation data that the effect of our approach is specific for the target gene of interest, *CDKL5*.

## MATERIALS AND METHODS

### Cloning of sgRNAs

For the cloning of *CDKL5* sgRNAs 20-bp spacer sequences were selected within ±1 kb of the *CDKL5* TSS (chrX:18 443 725, hg19) using the online tool CHOPCHOP (32). For transient transfection experiments, sgRNAs were cloned into a sgRNA expression vector (Addgene plasmid # 73797) following a previously published protocol (33). For transductions, sgRNAs were cloned into a lentiviral expression vector (Addgene plasmid # 73797) as previously described (34). Spacer sequences used to create target-specific sgRNA expression vectors are listed in Supplementary Table S1. All constructs were sequence confirmed by Sanger sequencing (Genewiz, Inc, South Plainfield, NJ, USA) and chromatograms were analysed using SnapGene software (from GSL Biotech; available at snapgene.com).

### Transient transfection experiments

U87MG (ATCC, Manassas, VA, USA) and Lenti-X 293T (Takara Bio USA, Inc., Mountain View, CA, USA) were grown in media containing high-glucose DMEM supplemented with 1% l-glutamine (Thermo Fisher Scientific, Waltham, MA, USA) and 10% HyClone heat-inactivated FBS (Thermo Fisher Scientific). BE(2)C (ATCC) cells were grown in DME/F12 (Thermo Fisher Scientific) supplemented with 1% l-glutamine and 10% HyClone heat-inactivated FBS. For gene expression modulation experiments, cells per well were grown to 80% confluency and transfected within 24 h of plating using Lipofectamine 3000 (Life Technologies) following the manufacturer's instructions with 3 ul of Lipofectamine 3000 reagent diluted in 500 ul Opti-MEM reduced serum media (Thermo Fisher Scientific). Transfections were performed in 12-well plates using either a mock-treatment (diluted transfection reagent) or 700 ng dCas9 expression vector (Fuw-dCas9-Tet1CD-P2A-BFP, Addgene plasmid #108245; Fuw-dCas9-Tet1CD_IM, Addgene plasmid # 84479; pLV hUbC-dCas9-T2A-GFP, Addgene plasmid # 53191; pLV hUbC-dCas9 VP64-T2A-GFP, Addgene plasmid # 53192) and 300 ng of equimolar pooled sgRNA expression vectors. Transfection medium was replaced 24 h post-transfection with complete growth medium. Forty-eight hours post-transfection, cells were rinsed in 1X DPBS (Thermo) and lysed in the well using TriZol (Ambion, Austin, TX, USA). Total RNA was extracted using the Direct-zol RNA Miniprep kit (Zymo Research, Irvine, CA, USA) and 500 ng RNA was reverse transcribed using RevertAid First Strand cDNA Synthesis Kit according to the manufacturer's instructions using random hexamer primers. Real-time PCR was performed in triplicate with 20 ng of cDNA per reaction and PowerUp SYBR Green Master Mix (Thermo Fisher Scientific) using the StepOne Plus Real Time PCR system (Thermo Fisher Scientific) and the StepOne Plus software was used to extract raw CT values. Gene expression analysis was performed with *GAPDH* as a reference gene in three biological replicates using exon-spanning primers for *CDKL5* and *GAPDH*. All primer oligonucleotides used in this study are listed in Supplementary Table S1. Fold change of gene expression was calculated as the delta delta CT between *GAPDH* and *CDKL5* transcript levels normalized to mock-treated relative *CDKL5* transcript levels as the reference.

### Integrative XCI status analysis of *CDKL5*

In order to determine the XCI status of *CDKL5*, we used publicly available data from GTEx (https://gtexportal.org) to determine the sex-biased expression using 27 GTEx v6p tissues and blood dendritic cells from a female of Asian ancestry (24A) to assess XCI status of *CDKL5* (16). We used publicly available microarray data to identify a single nucleotide polymorphism (SNP) in the *CDKL5* gene of SH-SHY5Y (35). We isolated genomic DNA from SH-SY5Y using the Quick-gDNA MiniPrep kit (Zymo Research). Total RNA was extracted using the Direct-zol RNA Miniprep kit (Zymo Research) and 500 ng RNA was reverse transcribed using RevertAid First Strand cDNA Synthesis Kit (Thermo Fisher Scientific). We confirmed the presence of the coding SNPs rs34567810 in *CDKL5* and rs1808 in the escape gene *CA5B* via Sanger sequencing (Genewiz, Inc) of both genomic DNA and RNA. Chromatograms were analyzed using SnapGene software (GSL Biotech).

### Lentivirus production and purification

To produce lentiviral particles as described before (36), a total of 50 million Lenti-X 293T cells were seeded into two T-225 flasks per viral packaging the day before transfection in high glucose DMEM supplemented with 10% fetal bovine serum and 1% l-glutamine. For each flask 25 μg of dCas9, dCas9-VP64, dCas9-TET1CD or sgRNA expression vector, 5 μg of pMD2.G (envelope, Addgene plasmid # 12259), and 25 μg of psPAX2 (gag/pol, Addgene plasmid #12260) were complexed with 140 ul using TransIT-293 (Mirus, Madison, WI, USA) according to the manufacturer's recommendation in Opti-MEM. Forty-eight hours after transfection, media was changed to 15mL of UltraCULTURE medium (Lonza, Basel, Switzerland). Vector supernatants were collected 72 hours post-transfection. Supernatant is initially centrifuged at 1500 rpm to clarify media and then concentrated by centrifugation at 3000 rpm using Centricon-Plus-70-Centrifugal-Filter-Units (MilliPoreSigma, Burlington, MA, USA). Viral aliquots were stored at –80°C. Virus for the expression of dCas9 effectors was titered by transduction of Lenti-X 293T cells and analyzed by flow cytometry for expression of GFP and BFP. All flow cytometry analyses were performed on the BD Fortessa at the UC Davis Flow Cytometry Shared Resource Core. Viral titers for the expression of sgRNAs were determined by using the qPCR lentivirus titration kit (Applied Biological Materials Inc., Richmond, BC). SH-SY5Y (ATCC) cells were grown in DME/F12 media containing 20% FBS and 1% l-glut. SH-SY5Y cells were seeded on six-well plates at a density of 300 000 cells per well and co-transduced with equimolar levels of dCas9 lentiviral particles equivalent to one Lenti-X 293T and a volume of dCas9 lentivirus equivalent to one Lenti-X 293T transducing unit and 5 × 10^7^ IU of each sgRNA expression vector in combination with 2.5 μg/ml protamine sulfate (Fresenius Kabi, Lake Zurick, IL). For cells co-transduced with dCas9-VP64 and dCas9-TET1CD lentiviral volumes equivalent to 0.5 Lenti-X 293T transducing units each were used. Cells were sorted 5 days post-transduction at passage 11 for expression of GFP and/or BFP using the Influx cell sorter at the UC Davis Flow Cytometry Shared Resource Core (Sacramento, CA, USA) and further expanded for 3–4 passages for subsequent analysis.

### Targeted X-reactivation analysis

SH-SY5Y cells from each FACS-isolated treatment group and unsorted cells were seeded at a density of 300 000 cells per well in six-well plates and allowed to grow until ∼70% confluency. Cells were then rinsed in 1× DPBS and lysed in the well using TriZol (Ambion). Total RNA was extracted using the Direct-zol RNA Miniprep kit (Zymo Research) and 500 ng RNA was reverse transcribed using RevertAid First Strand cDNA Synthesis Kit (Thermo Fisher Scientific). For X-reactivation analysis, 100 ng of cDNA from stable SH-SY5Y lines was used for PCR amplification using Phusion High Fidelity Mastermix (New England Biolabs, Ipswich, MA, USA). Each forward primer contained a unique 5-bp barcode sequence at the 5′ end for multiplexing (Supplementary Table S1). All amplicons were gel extracted and purified using the Zymo Gel DNA Recovery kit (Zymo Research) and pooled at equal concentrations for Illumina sequencing. Amplicon sequencing was performed by the CCIB DNA Core Facility at Massachusetts General Hospital (Cambridge, MA, USA). Forward and reverse reads of raw sequencing data were merged into a single long read using FLASH2 and barcodes were demultiplexed using FASTX at the beginning or end of the sequence read, allowing for a single mismatch each, yielding a mean read depth of >10 000 reads per sample. Processed FASTQ files were then analyzed for frequency of reads containing the reactivated C allele for the coding SNP rs35478150 identified in exon 16 of the *CDKL5* gene with the grep function over the total number of matched reads, yielding the reactivation frequency. Allele-specific RT-qPCR was performed as described above using a common forward primer and allele-specific reverse primers for the same coding SNP as analyzed by amplicon sequencing (Supplementary Table S1). Reactivation percentage was calculated as the percentage of relative Xi *CDKL5* expression over relative Xa *CDKL5* expression from mock-treated cells, normalized to *GAPDH*.

### Targeted DNA demethylation analysis

Genomic DNA from transduced and mock-treated cells was isolated using the Quick-gDNA MiniPrep kit (Zymo Research). Bisulfite conversion was performed using the EZ DNA Methylation-Gold Kit (Zymo Research) following the manufacturer's instructions. Primers for bisulfite-sequencing PCR were designed using MethPrimer with default settings (37) and unique 5-bp barcode sequences were added at the 5′ end for multiplexing (Supplementary Table S1). 100 ng of bisulfite converted DNA was used for PCR amplification with ZymoTaq polymerase (Zymo Research) and the 238-bp amplicon was purified with the QIAquick PCR Purification Kit (Qiagen, Hilden, Germany) and submitted for amplicon sequencing. Amplicon sequencing was performed by the CCIB DNA Core Facility at Massachusetts General Hospital (Cambridge, MA) and further processed as described above. Alignment of processed FASTQ files and read mapping to a 238 bp reference amplicon was performed using Bismark with default settings (38). Further analysis and methylation calling of sorted BAM files was performed using CGMapTools (39).

### Chromatin immunoprecipitation (ChIP) and ChIP-qPCR

ChIP was performed as previously described (40). Mock-treated and transduced cells were cross-linked 3–4 passages after FACS as described above in 1% formaldehyde for 10 min at room temperature and the reaction was stopped with 0.125 M glycine. Cross-linked cells were lysed with ChIP lysis buffer (5 mM PIPES pH 8, 85 mM KCl, 1% Igepal) with a protease inhibitor (PI) cocktail (Roche). Nuclei were collected by centrifugation at 2000 rpm for 5 min at 4°C and lysed in nuclei lysis buffer (50 mM Tris pH 8, 10 mM EDTA, 1% SDS) supplemented with PI cocktail. Chromatin was fragmented using the Bioruptor Pico (Diagenode, Denville, NJ, USA) and diluted with 5 volumes RIPA buffer (50 mM Tris pH 7.6, 150 mM NaCl, 1 mM EDTA pH8, 1% Igepal, 0.25% deoxycholic acid). ChIP enrichment was performed by incubation with 3 μg H3K27me3 antibody (ab6002, Abcam, Cambridge, UK) or 2 μg normal rabbit IgG (ab46540, Abcam) for 16 h at 4°C. Immune complexes were bound to 20 μl magnetic protein A/G beads (Biorad, Hercules, CA, USA) for 2 h at 4°C. Beads were washed 2 × with RIPA (Thermo Fisher Scientific) and 3  × with ChIP wash buffer (100 mM Tris pH 8, 500 mM LiCl, 1% deoxycholic acid). The final wash was performed in ChIP wash buffer with 150 mM NaCl. Cross-links were then reversed by heating beads in 100 μl ChIP elution buffer (50 mM NaHCO_3_, 1% SDS) overnight at 65°C, and DNA was purified using the QIAquick PCR Purification Kit (Qiagen, Hilden, Germany). ChIP-qPCR was performed with PowerUp SYBR Green Master Mix (Thermo Fisher Scientific) using the StepOne Plus Real Time PCR system (Thermo Fisher Scientific) and the StepOne Plus software was used to extract raw CT values. ChIP enrichment was calculated relative to input samples using the delta CT method.

### Whole-genome methylation analysis by Infinium MethylationEPIC array

Whole genome methylation analysis was performed following (40). Briefly, 300 000 cells for each treatment group were seeded in 6-well plates and allowed to grow to ∼70% confluency. Genomic DNA from transduced and mock-treated cells in biological duplicates was isolated using the Quick-gDNA MiniPrep kit (Zymo Research) and 500 ng submitted for bisulfite conversion and Illumina's Infinium MethylationEPIC BeadChip array by the Vincent J. Coates Genomics Sequencing Laboratory (Berkeley, CA, USA). The minfi package (41,42) was used to extract two channel raw data (RGChannelSet) from the IDAT files at the probe level for all 850,000 probes. The RGChannelSet was used for background subtraction using preprocessNoob (43) followed by preprocessFunnorm (44) to normalize the samples. Beta values for each site (beta = M/(M + U), where M and U denote the methylated and unmethylated signals) were extracted from the GenomicRatioSet, which is the data organized by the CpG locus level mapped to the genome. The ChAMP package (45) was used to filter probes using default settings with filterXY set to false. We then used the limma function within ChAMP (46,47) to detect differentially methylated positions at default settings and merged the output file with the individual FunNorm beta values. In order to determine differentially methylated promoter regions, CpG sites were selected for cgi.feat. TSS200-island and TSS1500-island and a mean difference in beta value of ±0.05. Differentially methylated genes were defined as genes with at least 3 differentially methylated positions in the promoter. Venn diagrams were generated using http://bioinformatics.psb.ugent.be/.

### RNA-Seq library preparation and analysis

Global changes to transcription were assessed using RNA-Seq. Briefly, 300 000 cells for each treatment group were seeded in six-well plates and allowed to grow to ∼70% confluency. Cells were then rinsed in 1× DPBS and lysed in the well using TriZol (Ambion). Total RNA was extracted using the Direct-zol RNA Miniprep kit (Zymo Research). RNA was quantified with Nanodrop and 1 ug of RNA was used for each library. RNA libraries were generated using the NEBNext Ultra II RNA Library Prep kit (NEB) following manufacturer's instructions. Libraries were multiplexed and pooled for a single lane of sequencing on a HiSeq4000. Sequencing reads were de-multiplexed and aligned to the Hg38 reference genome with STAR Universal Aligner version 2.5.3a using the following settings: *Indexed Reference Genome:* Ensembl reference genome and annotation files for Hg38 release 77 were downloaded and complied into a single file, Genome was indexed using the following arguments ‘STAR –runMode genomeGenerate –runThreadN 12 –genomeDir /STAR_INDEX_HG38 –genomeFastaFiles GRCh38_r77.all.fa –sjdbGTFfile Homo_sapiens.GRCh38.77.gtf –sjdbOverhang 149’; *Sample Read Alignment:* alignment of each sample's reads was performed with the following arguments: ‘STAR –runThreadN 24 –genomeDir /STAR_INDEX_HG38 –outFileNamePrefix /STAR/SampleName_ –outSAMtype BAM SortedByCoordinate –outWigType bedGraph –quantMode TranscriptomeSAM GeneCounts –readFilesCommand zcat –readFilesIn Sample-R1.fastq.gz Sample-R2.fastq.gz’. Differential Expression (DE) analysis was performed with DESeq2 (48) software in R Studio. First, gene count files were combined into a single file. Then, normalization and DE analysis were performed using a dCas9 control. DE gene lists from pairwise comparisons were exported into .csv files and utilized for GO term analysis using DAVID (https://david.ncifcrf.gov). Volcano plots were generated using ggplot2 software in R studio.

### Off-target analysis

Off-target analysis of CRISPR sgRNAs was performed using the CasOFFinder tool (http://www.rgenome.net/cas-offinder/) (49). Briefly, 20bp spacer sequences for the three top sgRNA candidates without PAM sequences were used as the query using hg38 as the reference genome for canonical SpCas9 PAM sites. The algorithm was executed using three or less mismatches and DNA and RNA bulge sizes of 1. In order to extend the list from off-target sites to potential off-target genes, we included genes in a ±5 kb window using the Table Browser function of the UCSC Genome Browser. The list of off-target genes was then overlapped with all differentially expressed genes from the three conditions as well as differentially methylated probes from the dCas9-TET1CD comparison with dCas9 catalytically inactive TET1.

### Statistical analysis

Statistical analyses were performed in Prism 8 (GraphPad Software, San Diego, CA, USA) and in R Studio 3.6.0. Statistics are presented as the mean ± SD. Targeted assessments were performed in biological triplicates. Genome-wide assessment were performed in triplicates unless otherwise noted. Between-group differences were analyzed using a One-way analysis of variance (ANOVA). When appropriate, a Tukey's post hoc test was performed. Statistical differences between the means of two groups were determined using an independent samples *t* test. The *P* value cut-off for all targeted analyses was set at 0.05 for all analyses. Statistical analyses of differentially methylated sites were performed using the limma function embedded in ChAMP in R Studio 3.6.0. The null hypothesis was rejected for tests with FDR <5%. Statistical analyses of differentially expressed genes was performed using DESeq2 in R Studio 3.6.0. The null hypothesis was rejected for tests with FDR <1%.

## RESULTS

### Programmable transcription of the *CDKL5* gene

**Figure 1.**
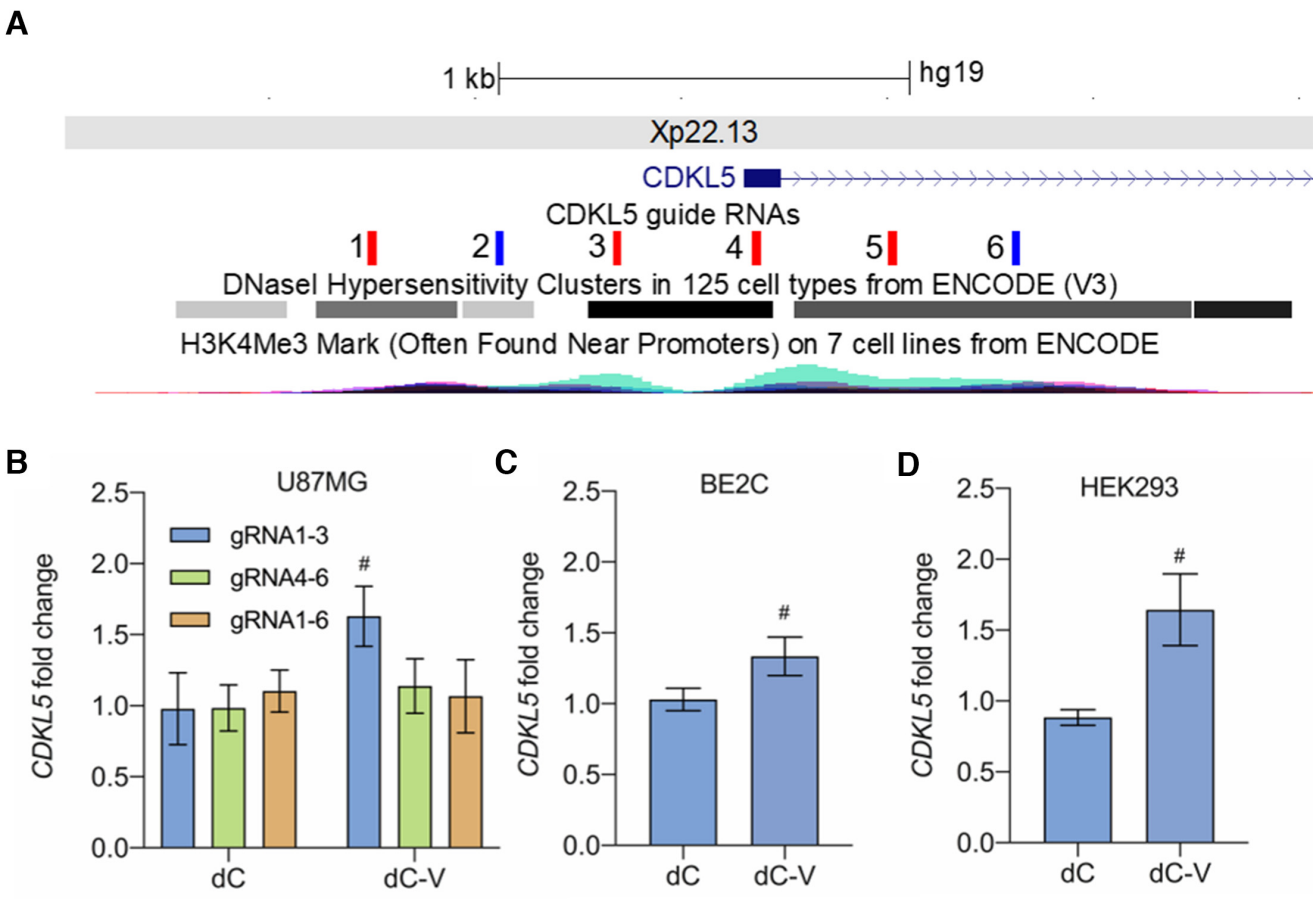
Programmable transcription of the *CDKL5* gene. (**A**) UCSC genome browser snapshot of the target sites of the six sgRNAs directed against the *CDKL5* promoter on Xp22.13. DNase hypersensitive sites and H3K4me3, often found near promoters are derived from ENCODE. Sense sgRNAs are shown in blue, antisense sgRNAs in red. (**B**) *CDKL5* mRNA fold change relative to mock-treated cells in U87MG cells determined by RT-qPCR resulting from programmable transcription using a dCas9-no effector (dC) or dCas9-VP64 (dC-V) in combination with different pools of three to six sgRNAs targeted to the *CDKL5* promoter 48 h after transient transfection. ^#^Significantly different from dCas9 sgRNAs 1–3, *n* = 3 independent experiments, Tukey's HSD, *P* < 0.05. (**C**) *CDKL5* mRNA fold change relative to mock-treated cells in BE2C determined by RT-qPCR resulting from programmable transcription using dCas9-no effector or dCas9-VP64 co-expressed with sgRNAs 1–3 48 h after transient transfection. (**D**) *CDKL5* mRNA fold change relative to mock-treated cells in Lenti-X 293T determined by RT-qPCR resulting from programmable transcription using dCas9-no effector or dCas9-VP64 co-expressed with sgRNAs 1–3 48 h after transient transfection. ^#^Significantly different from dCas9 sgRNAs 1–3, *n* = 3 independent experiments, Student's *t*-test *P* <0.05.

In order to investigate if the *CDKL5* gene is amenable for transcriptional reprogramming via dCas9 effector domains we transiently co-transfected U87MG cells with dCas9 constructs and gRNA expression vectors. We used a dCas9-VP64 expression plasmid (dC-V). Plasmid expressing dCas9 without effector domain was used as a control (dC). We designed six individual guide RNAs spanning DNase I hypersensitive sites and H3K4me3 peaks of the *CDKL5* promoter within a ±1 kb window on either side of the *CDKL5* transcriptional start site (Figure 1A). On the basis of previous observations that several guide RNAs are required for gene activation with dC-V (50), we tested several combinations of 3–6 guide RNAs (Figure 1B). We performed RT-qPCR and observed significant activation of *CDKL5* expression with the combination of guide RNAs 1–3 paired with dC-V targeting a region upstream of the transcriptional start site. *CDKL5* expression increased 1.6-fold in U87MG cells when compared to dC (*P* = 0.023). No significant difference between dC and dC-V was observed with cells transfected with guide RNAs 1–6 or guide RNAs 4–6. In concordance with U87MG cells, transfection with guide RNAs 1–3 and dC-V revealed a 1.3-fold and a 1.6-fold upregulation of *CDKL5* in BE2C cells (*P* = 0.0112, Figure 1C) and in HEK293 (*P* = 0.0424, Figure 1D), respectively, when compared to cells transfected with dC. Therefore, our results demonstrate the identification of a *cis*-regulatory element in the *CDKL5* promoter that allows for programmable transcription.

### dCas9-TET1CD significantly reactivates silenced *CDKL5* expression

Due to the lack of informative allele-specific polymorphisms in either of the previously tested cell lines we examined bi-allelic mRNA activation in female SH-SY5Y cells in order to assess whether the increase in gene expression was due to superactivation of the active *CDKL5* allele, reactivation of the silenced allele, or a combination of both. Data from a previously published comparative analysis across several GTEx tissues (16) demonstrate that *CDKL5* does not display female-biased expression, which serves as a proxy for XCI status when compared to the known escape gene *CA5B* (Supplementary Figure S1A). Analysis of XCI using pre-existing scRNA-seq data (16) to assess allele-specific expression from lymphoblastoid cells further revealed that *CDKL5* is monoallelically expressed only from the active X chromosome (Supplementary Figure S1B). In order to distinguish between the active and the inactive *CDKL5* allele, we confirmed the presence of a SNP (rs35478150) in the coding region of the *CDKL5* gene in SH-SY5Y cells. Sanger sequencing confirmed monoallelic expression of the active *CDKL5* A allele and silencing of the C allele. We also examined expression of a polymorphic site in *CA5B*, which showed bi-allelic expression from the active and escape allele (Supplementary Figure S1C).

Due to the importance of methylated CGI promoters in XCI, we investigated the role of a dCas9-TET1CD fusion protein for DNA methylation editing (dC-T). In order to determine X chromosome reactivation efficiency, we evaluated allele-specific activation facilitated by dC-V or dC-T. SH-SY5Y cells were transduced with lentiviral particles encoding the dC fusion proteins and the three guide RNAs. dCas9 expression plasmids also encode in-frame fluorescent markers GFP (dC and dC-V) or BFP (dC-T). Three days following transduction, transduced cells were selected by FACS based on the respective fluorescent marker (Supplementary Figure S2A).

**Figure 2.**
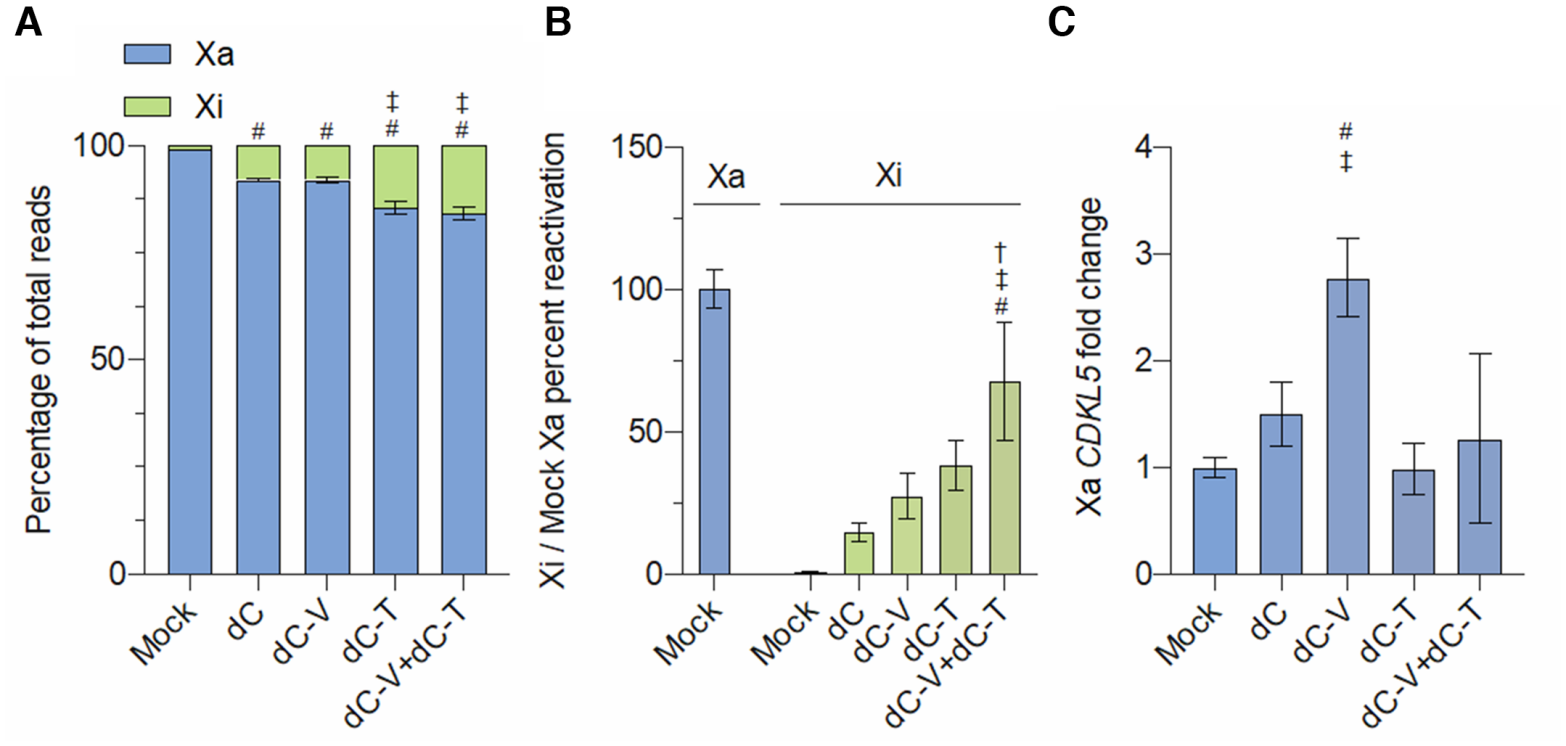
Targeted reactivation of *CDKL5* from the inactive X allele. (**A**) Allele specific read counts for the mRNA expression of the active (Xa) or inactive (Xi) *CDKL5* allele of mock-treated SH-SY5Y or after constitutive expression of dCas9 effector domains dCas9 (dC), dCas9-VP64 (dC-V), dCas9-TET1CD (dC-T) or a combination of dCas9-VP64 and dCas9-TET1CD (dC-V+dC-T) and sgRNAs 1–3 after 21 days post-transduction. ^#^Significantly different from mock-treated, ^‡^significantly different from dCas9, *n* = 3 independent experiments, Tukey's HSD, *P* < 0.05. (**B**) Relative Xi *CDKL5* mRNA expression of mock-treated or stably transduced SH-SY5Y relative to *CDKL5* Xa mRNA expression of mock-treated cells as determined by allele-specific RT-qPCR after 21 days post-transduction. ^#^Significantly different from dC, ^‡^significantly different from dC-V, ^†^significantly different from dC-T, *n* = 3 independent experiments, Tukey's HSD, *P* < 0.05 (**C**) Relative Xa *CDKL5* mRNA expression in mock-treated and stably transduced SH-SY5Y cells determined by allele-specific RT-qPCR after 21 days post-transduction. ^#^Significantly different from mock-treated, ^‡^significantly different from dCas9, *n* = 3 independent experiments, Tukey's HSD, all *P* < 0.05.

To determine reactivation of the silenced *CDKL5* allele with high sensitivity, we performed amplicon-based targeted RNA-sequencing (Figure 2A). Targeting of dC to *CDKL5* was sufficient to significantly reactivate expression of the silenced allele by >11-fold to 8% of total allelic reads compared to mock-treated cells (*P* < 0.0001). Transcriptional reprogramming using dC-V targeted to the *CDKL5* promoter did not show a significant increase when compared to dC. Strikingly, cells transduced with dC-T showed a 20.7-fold increase when compared to mock (*P*<0.0001) and a significant 1.8-fold increase above dC (*P* < 0.0001), leading to reactivation levels of up to 14.5% of total expression. Since dC-V has been shown to increase total *CDKL5* mRNA in other cell lines tested, we sought to determine if multiplexing dCas9-VP64 and dCas9-TET1CD (dC-T+dC-V) to the same locus further potentiates *CDKL5* reactivation. However, no significant difference was observed between dC-T and co-transduction of dC-T+dC-V in increasing the proportion of allelic reads derived from the inactive allele.

Due to the fact that the observed allelic reads via amplicon sequencing are a ratio of active versus silenced *CDKL5* expression, we performed allele-specific RT-qPCR in order to compare reactivation levels from the inactive allele to the active allele baseline expression in SH-SY5Y (Figure 2B). Similar to our amplicon sequencing data, we did not identify expression of the inactive allele above background (<1% Xi/Xa mock). We observed expression levels in cells transduced with dC of 14.8% Xi/Xa mock in cells treated with dC. No statistically significant increase in Xi/Xa mock expression over dC was observed in cells treated with dC-V (27.3%) or dC-T (38.2%). However, SH-SY5Y cells that were co-transduced with dC-V+dC-T showed a statistically significant increase of reactivation from the inactive allele (67.4%) when compared to dC (*P* = 0.0004), dC-V (*P* = 0.042) and dC-T (*P* = 0.038). These findings suggest that SH-SY5Y that have been treated with the dCas9 effector domains reach close to equal bi-allelic expression due to a synergistic effect of dC-V and dC-T on the previously silent, reactivated allele.

For the expression of the active *CDKL5* allele, we observed no significant difference between dC and mock-treated cells (Figure 2C). Moreover, we identified that dC-V significantly upregulates mRNA expression from the active allele by 3.0-fold when compared to mock (*P* = 0.0052) or 2.7-fold when compared to dC (*P* = 0.0073). No significant difference was observed between mock cells versus dC-T or dC-V+dC-T and dC versus dC-T or dC-V+dC-T. Noticeably, targeting dC-T to the *CDKL5* promoter did not significantly modulate active *CDKL5* mRNA levels.

### dCT significantly reduces DNA methylation

**Figure 3.**
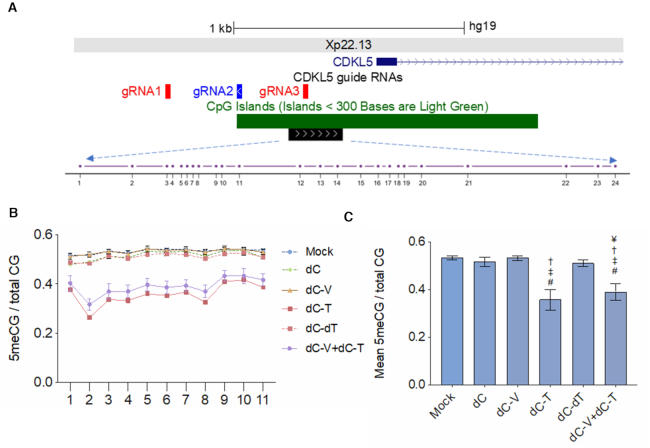
dCas9-TET1CD causes removal of DNA methylation from the *CDKL5* CGI promoter. (**A**) UCSC genome browser snapshot of the target sites of sgRNAs 1–3 directed against the *CDKL5* promoter on Xp22.13 and a large CpG Island (>1 kb) spanning the transcriptional start site of *CDKL5*. The black box represents a >200 bp region assessed for targeted DNA methylation changes containing 24 individual CpG dinucleotides (drawn to scale). (**B**) 5-methylcytosine levels in a CpG context (5meCG) over total CpG context as assessed by targeted bisulfite sequencing across 11 CpG dinucleotides in mock-treated cells or cells transduced to constitutively express dCas9-no effector (dC) or dCas9 fused to either VP64 (dC-V) or TET1CD (dC-T), a combination thereof (dC-V+dC-T) or a catalytically inactive TET1CD (dC-dT). X-axis depicts the individual CpG position relative to the amplicon (not drawn to scale). (**C**) Mean 5-methylcytosine levels in a CpG context over all 11 CpG dinucleotides in all treatment groups. ^#^Significantly different from mock-treated cells, ^‡^significantly different from dCas9, ^†^significantly different from dC-dT, ^¥^significantly different from dC-T, *n* = 3 independent experiments, Tukey's HSD, all *P* < 0.05.

The status of XCI is highly correlated to promoter CGI methylation (12,20). Due to the differences in targeted reactivation between effector domains, we performed targeted bisulfite amplicon sequencing in the *CDKL5* core promoter region in order to identify the role of differential DNA methylation in X-reactivation between groups (Figure 3A). We generated PCR-based amplicons that allowed us to measure the ratio of 5-meCG/total CG at 24 CpG individual dinucleotides in the *CDKL5* core promoter by deep sequencing (Figure 3B, Supplementary Figure S3). Due to the lack of a polymorphism in the promoter region, we assessed biallelic CpG methylation, assuming that DNA methylation was primarily present on the XCI silenced allele. We observed two segments of DNA methylation that were demarcated by a dip in methylation at CpG dinucleotide position 12. The first segment showed that the *CDKL5* promoter was partially methylated in mock-treated SH-SY5Y (53% 5-meCG/CG ± 0.9%, Figure 3B), while the second segment showed a decreased baseline DNA methylation level and more variability of 5-meCG/CG (25.4% 5-meCG/CG ± 16.8%, Supplementary Figure S3), suggesting that partial methylation of the core region containing the first 11 CpGs is critical for regulation of *CDKL5* transcription. Amplicon sequencing of bisulfite converted genomic DNA revealed the mean 5-meCG/total CG ratio across the first 11 CpG sites was 53.3% in mock and 51.6% in dC transduced cells (Figure 3C). Strikingly, we observed a 17.5% decrease of 5-meCG/CG in cells transduced with dC-T compared to mock-treated cells (*P* < 0.0001) and a 15.9% decrease to dC (*P*<0.0001). We demonstrate that this heterogeneous response on the bulk level is due to the catalytic activity of dC-T, since a catalytically inactive TET1 mutant (dC-dT) fails to disrupt methylation at 51% 5-meCG/CG (*P* < 0.0001). The combinatorial treatment of dC-T+dC-V also showed a significant reduction in methylation levels of 14.3% compared to mock-treated cells (*P* < 0.0001) and 12.6% compared to dC (*P* < 0.0001). However, the combination of dC-T and dC-V had significantly higher levels of methylation when compared to dC-T alone. Interestingly, the combination of dC-T and dC-V had the greatest increase of the inactive allele. This may be due to TET1 achieving a level of demethylation that allows for gene transcription. In fact, the addition of dC-V significantly decreased the amount of demethylation, indicating, that dC-V does not contribute to DNA demethylation as a mechanism of transcriptional activation.

### Targeted loss of repressive H3K27me3 in the *CDKL5* promoter

**Figure 4.**
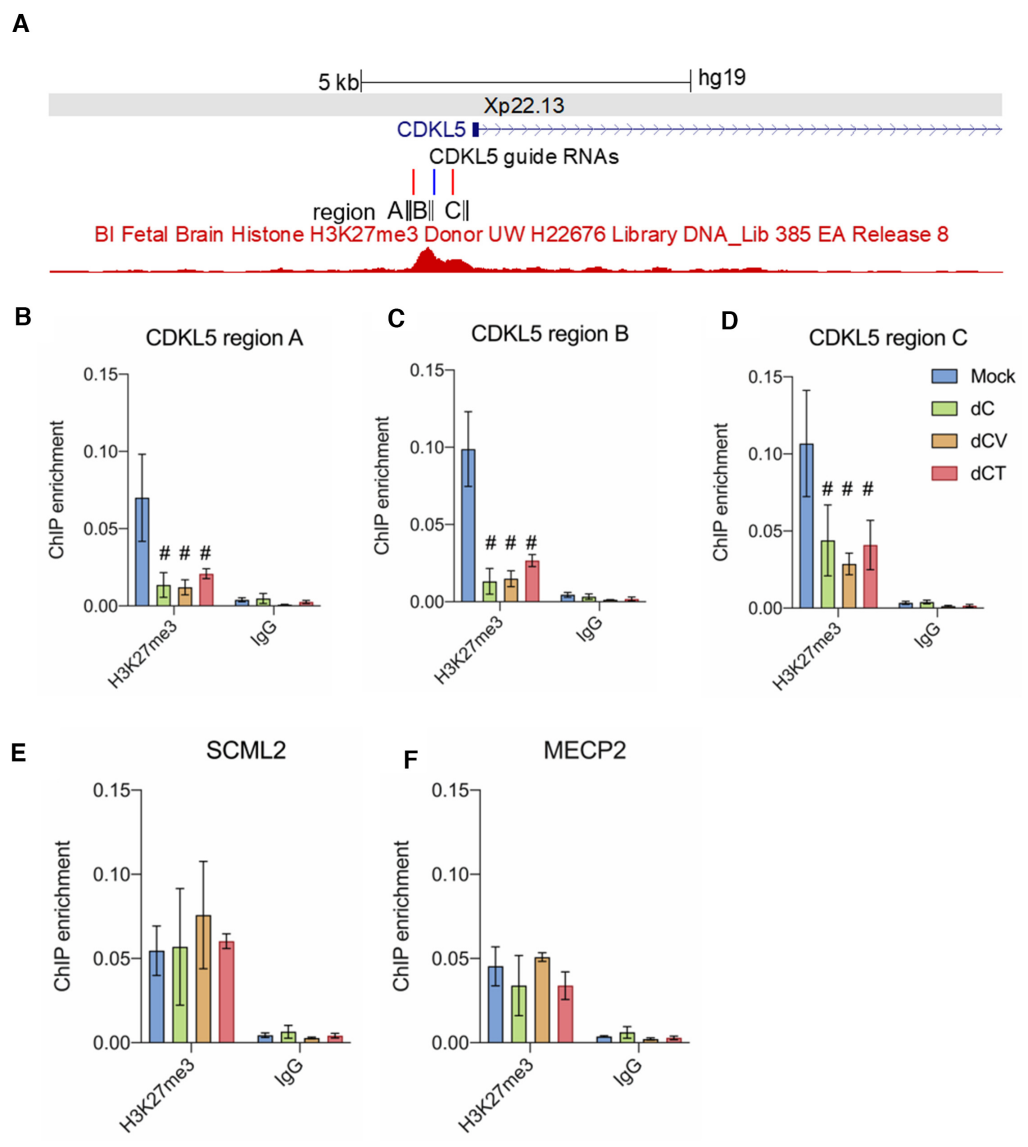
Depletion of the XCI hallmark histone modification H3K27me3. (**A**) UCSC genome browser snapshot of the target sites of sgRNAs 1–3 directed against the *CDKL5* promoter on Xp22.13 and H3K27me3 peaks derived from ENCODE. Black boxes show the regions assessed by ChIP-qPCR. (**B**) Input normalized H3K27me enrichment levels determined by ChIP-qPCR in region A of the *CDKL5* promoter in mock-treated cells or cells transduced to constitutively express dCas9-no effector (dC) or dCas9 fused to either VP64 (dC-V) or TET1CD (dC-T). (**C**) Input normalized H3K27me enrichment levels determined by ChIP-qPCR in region B of the *CDKL5* promoter. (**D**) Input normalized H3K27me enrichment levels determined by ChIP-qPCR in region C of the *CDKL5* promoter. (**E**) Input normalized H3K27me enrichment levels determined by ChIP-qPCR in the promoter of the nearest neighboring gene promoter, *SCML2*. (**F**) Input normalized H3K27me enrichment levels determined by ChIP-qPCR in the promoter of a distal gene, *MECP2*, that serves as a negative control. ^#^Significantly different from mock-treated cells, *n* = 3 independent experiments, *P* < 0.05.

As previously demonstrated, genes that escape from XCI show a specific epigenetic signature, such as the depletion of the repressive histone mark H3K27me3. Therefore, we sought to investigate whether targeted reactivation of the observed allele coincided with a remodelling of heterochromatin. We used ChIP-qPCR to test three different regions within a 1-kb fragment upstream of the transcriptional start site for changes in the H3K27me3 mark that have strong signal enrichment in brain tissue as determined by ENCODE and overlap the guide RNA target sites (Figure 4A). When compared to mock-treated cells, treatment with dC by itself depleted H3K27me3 signal 3.5-fold in region A (*P* = 0.0073, Figure 4B); 2.9-fold in region B (*P* = 0.0002, Figure 4C) and 1.5-fold in region C (*P* = 0.00453, Figure 4D). There was no significant difference between treatment with dC, dC-V or dC-T. In order to understand the effect of histone-based feedback and spreading of the depletion of the histone mark across neighboring nucleosomes in our treated cells, we investigated the signal of H3K27me3 in distal neighboring regulatory regions. We performed ChIP-qPCR on the nearest neighboring gene to the *CDKL5* promoter (–70 kb) and tested for H3K27me3 signal (Figure 4E). There were no significant differences between groups for H3K27me3 signal in the promoter region of the *SCML2* gene. Therefore, it is suggestive that the loss of H3K27me3 remains confined to the target site and is associated with gene reactivation. Finally, no significant difference between groups was observed for H3K27me3 signal at an unrelated negative control region in the *MECP2* promoter on the long arm of the X chromosome (Figure 4F).

### dC-T transduced cells show global promoter hypomethylation

**Figure 5.**
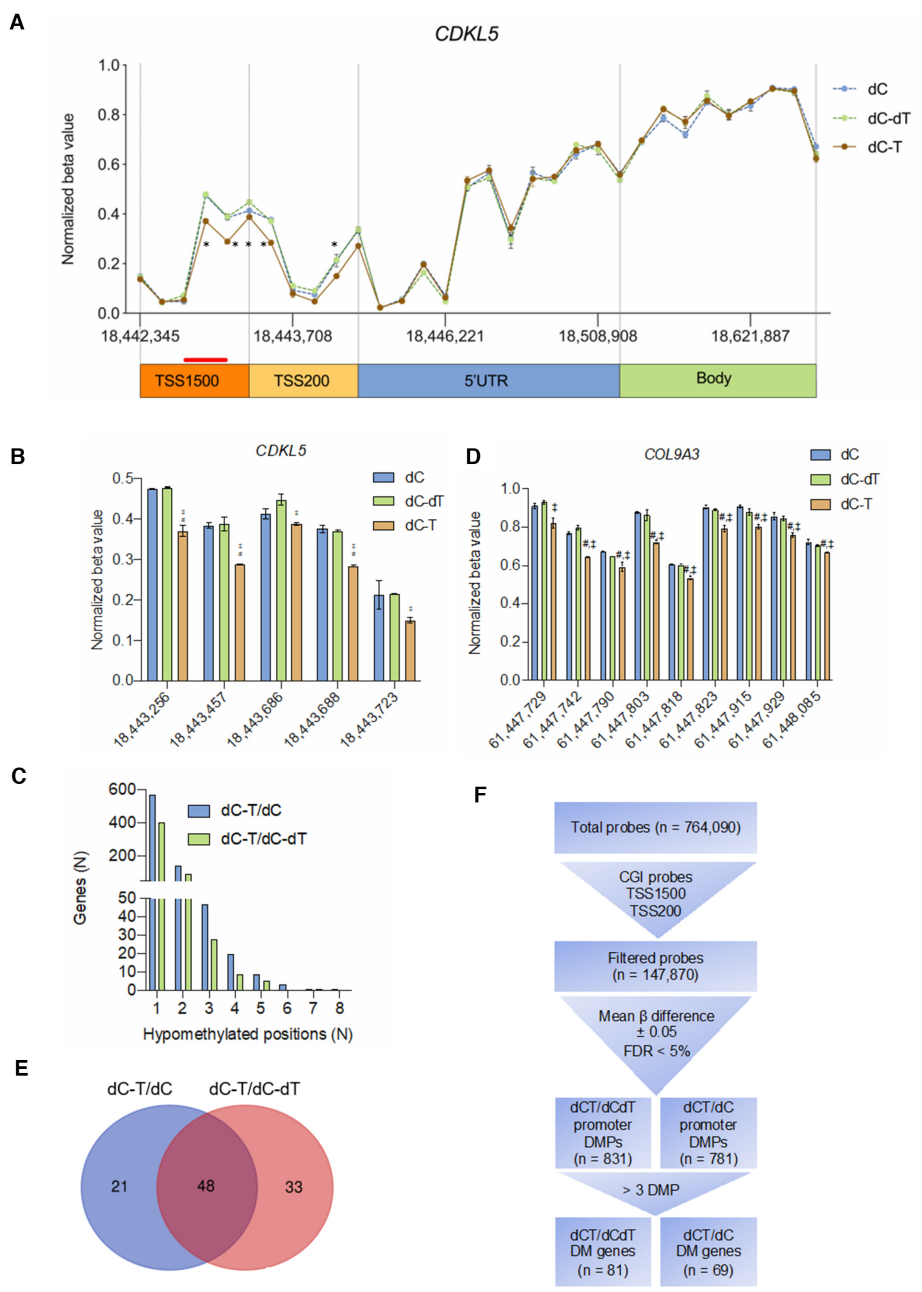
Global DNA hypomethylation due to constitutive dCas9-TET1CD expression. (**A**) Thirty-two CpG positions shown with their respective location on the X-chromosome (hg19) from the 850K MethylationEPIC array across the *CDKL5* promoter were used to assess gene-wide changes in DNA methylation levels represented as changes in the beta value of the TSS200, TSS1500, 5′UTR and gene body of *CDKL5*. After transduction with dCas9-no effector (dC), dCas9-TET1CD (dC-T) and a catalytically inactive TET1CD (dC-dT), we found reduced DNA methylation levels in the TSS1500 and TSS200 region of cells transduced with dCas9-TET1CD. The red line demonstrates the sgRNA binding sites in the *CDKL5* promoter. *Significantly differentially methylated positions for further assessment. (**B**) Side-by-side assessment of significantly differentially methylated positions in the *CDKL5* promoter with a mean difference in beta value of <0.05. ^#^Significantly different from dC, ^†^significantly different from dC-dT, *n* = 2 independent experiments, FDR < 5%. (**C**) Histogram of the number of genes by the number of significantly hypomethylated sites of dCas9-TET1CD transduced cells when compared to dCas9 or a catalytically inactive TET1 fused to dCas9 demonstrates that the majority of genes shows only a single probe falling within the respective promoter region. (**D**) Side-by-side assessment of significantly differentially methylated positions in the *COL9A3* promoter with a mean difference in beta value of <0.05. ^#^Significantly different from dC, ^†^significantly different from dC-dT, *n* = 2 independent experiments, FDR < 5%. (**E**) Venn diagram of shared genes between dCas9-TET1CD comparisons with dCas9 or a catalytically inactive TET1CD mutant shows an overlap of 48 genes between the two groups. (**F**) A flow chart diagram representing the analysis pipeline for genome-wide methylation effects of dCas9-TET1CD, starting from a total number of probes, down to significantly differentially methylated sites and ultimately differentially methylated genes.

In order to determine on- and off-target effects of dC-T on the DNA methylome in stably transduced SH-SY5Y cells, we used the Illumina Infinium HumanMethylationEPIC (EPIC) array to interrogate 764 090 CpG sites genome-wide (Supplementary Table S2, Figure S4). We identified a smaller subset of 147 870 probes (19.4%) within CpG islands. Out of this subset, we further enriched for 59 264 probes that were found within 1500 and 200 bp of the TSS. Understanding the role of dCas9 binding to distal regulatory elements was outside of the scope of the analysis. In our pairwise comparisons to determine differentially methylated (DM) positions between dC-T and dC-dT or dC, we set a cut-off of mean difference in beta value greater than 0.05 (FDR < 5%). In order to validate our criteria, we first analyzed all 32 methyl-probes mapping to the *CDKL5* gene without filtering for probe features (Figure 5A and B). In concordance with our targeted bisulfite approach, we identified partial methylation of the promoter region of control treated cells (dC, dC-dT), which was modestly but significantly reduced near the sgRNA target site (Figure 5A, red line) in cells transduced with dC-T. We did not identify DM positions in any of the other genic features, which further demonstrates the highly distinctive role of DNA methylation signatures in CGI promoters. No DM positions were identified in the nearest neighboring gene to the *CDKL5* promoter (*SCML2*) once again confirming that the dC-T-induced DNA demethylation is targeted to the *CDKL5* promoter. In total, we identified 795 or 747 differentially hypomethylated promoters and 34 or 26 differentially hypermethylated promoters for the comparison between dC-T and dC or dC-dT, respectively. Due to the small number of differentially hypermethylated sites and the fact that gene repression due to off-target hypermethylation of TET1CD is unlikely, we omitted hypermethylated sites from the further analysis. The majority of differentially hypomethylated sites in gene promoters due to the introduction of dC-T showed only a single DM position (568 genes when compared to dC, 402 genes when compared to dC-dT), likely not eliciting an effect on transcription (51) (Figure 5C). Since *CDKL5* had at least 3 differentially hypomethylated sites, we only considered genes with at least three DM sites as a differentially hypomethylated gene promoter. The gene with the highest number of DM positions was *COL9A3*, showing eight hypomethylated sites within the promoter (Figure 5D). We identified a total of 69 or 81 genes when compared to dC or dC-dT respectively. Forty-eight genes were conserved between the pairwise comparisons (Figure 5E and F).

### Specificity of *CDKL5* sgRNAs and dCas9 effector domains

**Figure 6.**
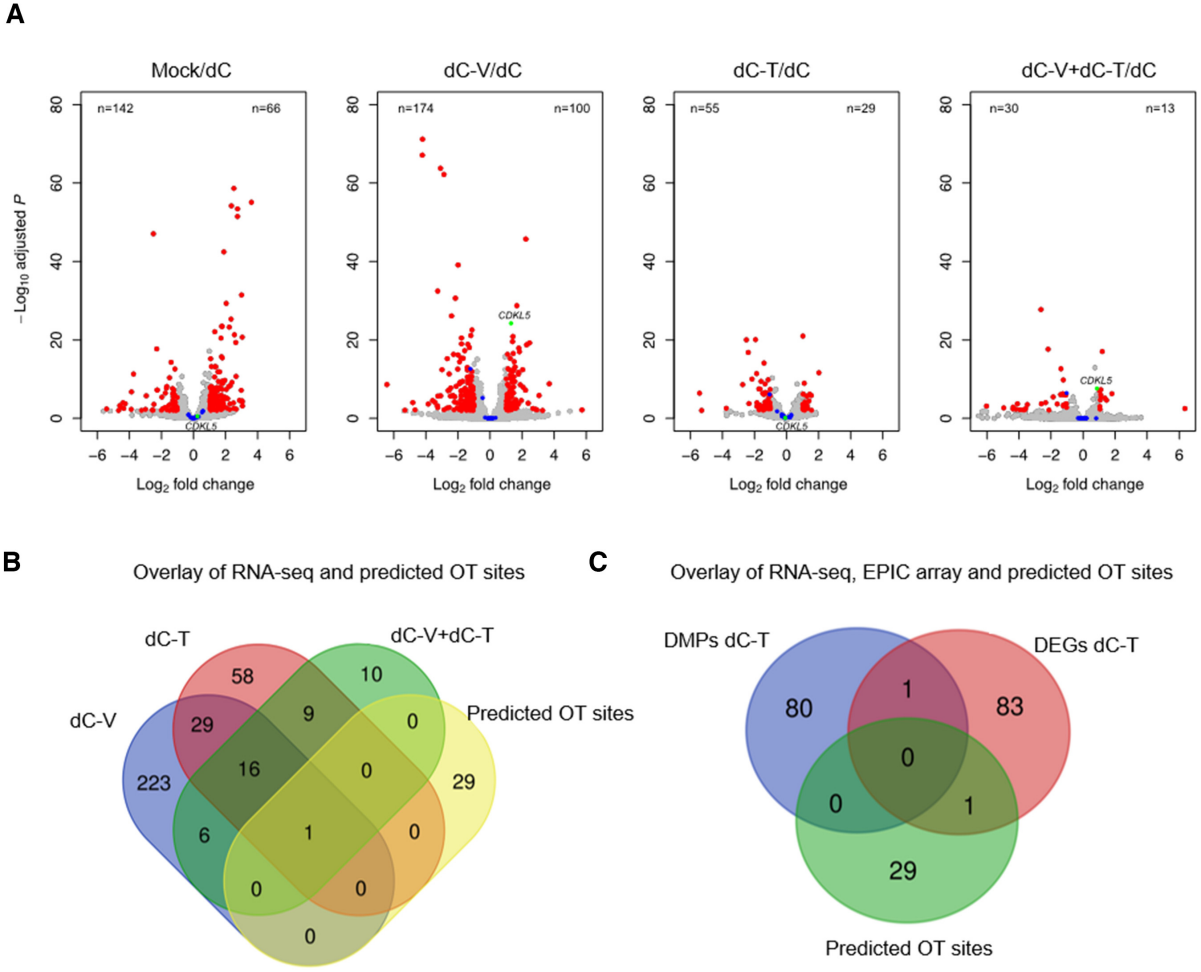
Off-target analysis of CRISPR/dCas9 effectors by RNA-seq. (**A**) Volcano plot of significance (FDR adjusted *P* value) versus fold change for differential DESeq2 expression analysis of mock-treated, dCas9-VP64 (dC-V), dCas9-TET1CD (dC-T) or dCas9-VP64 and dCas9-TET1CD (dC-V+dC-T) guided by sgRNAs 1–3 to the CDKL5 promoter compared to a dCas9-no effector control (dC). Differentially expressed genes are highlighted in red (FDR < 1%, log fold change >1), predicted CRISPR off-target sites are highlighted in blue and the *CDKL5* target gene is highlighted in green. The number of downregulated genes is found in the upper left of each panel, the number of upregulated genes is found in the upper right of each panel. (**B**) Venn diagram showing the overlap of differentially expressed genes between all conditions and the putative off-target list. A single gene, *CNTNAP2* is shared between all four groups as a putative off-target. (**C**) Venn diagram showing the overlap between differentially expressed genes and differentially methylated positions identified in a comparison between dCas9-TET1CD and dCas9 and potential CRISPR off-targets.

To evaluate the effect of targeting *CDKL5* with dCas9 effector fusions on global gene expression we performed RNA-seq in stably transduced SH-SY5Y. We observed that the introduction of dC alone causes 208 differentially expressed (DE) genes when compared to mock-treated cells, likely due to the introduction of the lentiviral machinery (66 up- and 142 downregulated genes, Figure 6A, Supplementary Table S3). Therefore, we performed pairwise comparisons with dC as the control. When compared to cells transduced with dC, we found that cells transduced with dC-V or dC-V+dC-T targeted to *CDKL5* specifically increase *CDKL5* expression without altering expression of adjacent transcripts (nearest neighboring gene upstream and downstream of *CDKL5*). No significant upregulation of *CDKL5* was detected in cells treated with dCas9-TET1CD. We identified four genes containing heterozygous SNPs in the coding region within a ±2 Mb range of the *CDKL5* target site (*MAP3K15*, *RAI2*, *NHS* and *BEND2*, Supplementary Table S3). However, mean read counts for these genes were generally unchanged from the mock-treated group, albeit 3 out of 4 genes were lowly or not expressed. Regardless, since the mean read counts for these gene were not significantly altered, it is suggestive that the X-chromosomal genes were not reactivated. In total, we identified 274 differentially expressed (DE) genes in dC-V (100 up- and 174 downregulated genes), 84 DE genes in dC-T (*n* = 29 up- and *n* = 55 downregulated) and 43 DE genes in dC-V+dC-T (13 up- and 30 downregulated genes). In general, we observed a greater number of differentially downregulated genes in transduced cells, which we do not attribute to off-target binding of the constructs as both effector domains confer transcriptional activation to direct targets, not repression.

Although *CDKL5* sgRNA sequences were designed to target a unique site in the human genome, it is possible that our sgRNAs can tolerate mismatches leading to off-target binding. In order to address this issue, we searched for potential off-target (OT) sites with up to three mismatches within the sgRNA sequences using CasOFF-Finder, which scans for both nucleotide mismatches and bulges in the sequence, thereby making it a comprehensive *in silico* prediction tool for OT analysis (49). In order to include OT sites that fall within intergenic regions, we extended the targets by ±5 kb from the predicted OT site to include neighboring transcripts and identified a total of 30 predicted OT genes (Supplementary Table S3). Strikingly, the majority of OT sites required at least two mismatches, with sgRNA 2 only being permissive for OT sites with three mismatches in the sequence. Out of 30 OT genes, we identified a single target, *CNTNAP2*, that was downregulated in dC-V, dC-T and dC-V+dC-T in all three conditions. While the predicted OT site for *CNTNAP2* falls within an intronic sequence of the gene, we cannot preclude that the differential expression is a consequence of off-target binding of the dCas9 effector domain. Cells transduced with dC-V showed the highest number of unique differentially expressed transcripts (*n* = 223), followed by dC-T (*n* = 58) and dC-V+dC-T (*n* = 10). A total of 16 differentially expressed genes were shared between the three conditions (Figure 6B). A gene ontology analysis did not reveal significant enrichment of terms, indicating that the set of DE genes does not share a common pathway (Supplementary Table S3).

Next, we sought to assess whether the observed global changes in DNA methylation in cells transduced with dC-T were associated with altered transcript levels. We investigated the overlap between all 81 differentially hypomethylated genes in CGI promoter regions with greater than three DM positions, all 84 DE genes and the predicted 30 OT genes (Figure 6C). Overall, we identified that a single gene (*HHIPL1*) showed association between differentially methylation and expression (Supplementary Figure S5). Finally, we did not identify genes overlapping the OT genes and DM positions. This data suggests that the observed global DNA hypomethylation of promoters poorly correlates with gene expression.

## DISCUSSION

A significant number of X-linked genes escape XCI and are expressed from the inactive X chromosome (16). Whether or not the epigenetic signature associated with these escapees is a cause or merely a consequence of expression from otherwise transcriptionally inert X-chromatin remains to be elucidated (17). Here, we demonstrate for one such epigenetic barrier in a specific gene context, that removal of CGI methylation from the promoter of the X-chromosomal gene *CDKL5* by directing a fusion of the catalytic domain of TET1 to dCas9 results in reactivation of gene expression in a targeted manner. In addition, employment of a strong transcriptional activator further increases the degree of escape in a synergistic fashion, resulting in expression levels in excess of 60% of the inactive allele when compared to the active allele.

We demonstrate that programmable transcription using a transactivator achieves a moderate but significant *CDKL5* upregulation when compared to other reported CRISPRa target sites (52) that was achieved across several cell lines. However, the effect of the VP64 transactivator was mainly due to superactivation of the already active allele, demonstrating that the epigenetic landscape of active X-chromatin presents a chromatin state more permissive for programmable transcription. Unexpectedly, we identified that binding of dCas9 with no effector was capable of reactivating *CDKL5* expression from the silent allele. This may be due to the large dCas9 protein serving as a pioneer factor when constitutively expressed and targeted to transcriptionally inactive X-chromatin, thereby causing limited gene reactivation on its own in a gene and chromatin specific context. In contrast to other studies (53,54), we did not observe hindrance of dCas9 binding to regions largely embedded in CpG-dense hypermethylated CGI promoters. We cannot rule out, however, that binding of a sgRNA outside of the methylated region on the inactive X chromosome is, at least in part, causative for the observed effect. This limited but significant reactivation was associated with the loss of the repressive histone mark H3K27me3 in the core promoter of *CDKL5*. While the direct role between dCas9 binding and depletion of the histone mark is not well understood, it is possible that binding of dCas9 causes displacement of the nucleosome, resulting in the loss of H3K27me3 and enhanced chromatin accessibility (55,56). In this study we assessed H3K27me3 due to its role in XCI. However, future studies to investigate dCas9 effect on nucleosome rearrangement would require the assessment of multiple histone subunits. In line with previous findings (2), we did not observe a spread of heterochromatin loss to the nearest neighboring gene, suggesting a targeted effect of dCas9 binding.

Previous studies suggested that nucleosome occupancy strongly impedes binding of (d)Cas9 (57,58). However, considering that our sgRNA design takes DNase hypersensitive sites into account, and considering the finding that the inactive X-allele is ∼1.2-fold more compact than the active allele (59), we demonstrate that a promoter of a gene on the inactive X-chromatin is generally targetable by dCas9. In addition, the accessibility of *CDKL5* can be further attributed to the location of the gene on a chromosomal segment that is part of a younger evolutionary strata of the X chromosome (17). Indeed, the majority of facultative and constitutive escape genes are located on the short arm of the X chromosome (17). Therefore, the chromosomal location of *CDKL5* might be favorable to induce an artificial escape. The fusion of VP64 to dCas9 did not further increase the observed reactivation, further supporting a steric effect primarily attributed to the large size of dCas9 that is not augmented by the addition of a small transactivator. The indirect recruitment of transcription factors by VP64 did not result in higher reactivation levels and may be due to the chromatin microenvironment, specifically the presence of DNA methylation as an epigenetic barrier that does not permit abundant transcription via VP64.

Changing the chromatin microenvironment via the introduction of TET1CD resulted in decreased DNA methylation of ∼15% in the *CDKL5* core promoter and significantly reactivated XCI-silenced *CDKL5*, thereby creating an artificial escape gene as previously defined at expression levels of at least 10% of the active allele (17). While we did not directly assess the levels of the 5-hydromethylcytosine intermediate, we demonstrate that targeted removal of 5-methylcytosine is mediated by the dioxygenase function of TET1CD. Likely due to the depletion of 5-methylcytosine substrate in the promoter of the active allele, recruitment of TET1CD to this region did not result in superactivation of the allele on the active X chromosome. Due to a lack of polymorphisms in the *CDKL5* promoter of SH-SY5Y cells, our model system did not allow us to test for allele specific changes of the epigenetic signature but was reliant on the assessment of total changes in DNA methylation in light of the fact that CGI methylation is highly correlative with the inactive X allele. Furthermore, a recent genome-wide assessment revealed global DNA hypomethylation of CGI promoters following TET1CD overexpression via lentiviral integration (29). However, our genome-wide assessment of promoter regions did not identify a strong correlation between reduced methylation of CpG sites and changes in transcription by RNA-seq. This is likely because the vast majority of genes identified only contain a single differentially methylated site indicative of one CpG dinucleotide, and the generally small effect size of the measured changes of DNA methylation. The change of a single CpG site in a promoter which typically contains multiple CpG sites likely would not result in biological significance, thus the lack of correlation with transcriptional activation. Since CGI promoters on the inactive X allele frequently show higher methylation levels than on the active X allele, targeted reduction of CpG methylation is directed to a single allele, unlike the case for autosomal genes. For example, in an autosomal setting, directed epigenetic editing may confer small changes to methylation levels of both alleles. These small changes do not necessarily translate to an additive effect on transcription if neither of the alleles reaches a threshold of biological significance. However, targeting a single X-chromosomal allele of a gene has the potential to concentrate the effects of epigenetic editing that would otherwise be divided over two alleles, increasing its potential to pass this arbitrary biological threshold. Thus, a decrease of DNA methylation on the inactive X chromosome can have a broader implication for regulation of gene expression. In future studies, the role of dCas9-TET1CD binding to putative distal enhancers that may result in differential methylation or gene expression will be assessed. In addition, it will be crucial to test whether a more transient delivery of TET1CD impacts the amount of observed methylation changes. While it was suggested that the effects of dCas9-TET1CD are specific (26), we and others demonstrate global DNA methylome changes (29). Similar findings have been demonstrated for genome-wide DNA methylation changes with fusions of the DNA methyltransferase DNMT3A to dCas9 (60), likely attributed to the high substrate abundance of methylated cytosines for constitutively expressed nuclear TET1CD not bound to the target site. This highlights the need to assess transient exposure of dCas9-effectors to the *CDKL5* promoter in order to reduce potential off-target effects in future studies.

Due to the strong effect of VP64 on upregulating genes in an unmethylated chromatin context, we assessed a combination of TET1CD and VP64 targeted to *CDKL5* via dCas9. We found a synergistic effect between removal of DNA methylation and strong transcriptional activation that resulted in a greater than 60% expression from the inactive allele. Since the employment of VP64 alone does not significantly increase reactivation levels, we believe that the introduction of dCas9-TET1CD causes a dynamic reprogramming in which methyl groups are removed from CpG dinucleotides, thus allowing for further binding of transcription factors to the inactive chromatin via an indirect recruitment from VP64. These findings support a synergistic effect between TET1CD and transactivators that have recently been supported by others (28,29). In future studies, the effect of improved transcriptional activators, such as the VP64–p65–Rta tripartite fusion (61) or the use of the SunTag (62) system will be harnessed to further potentiate the expression of XCI silenced *CDKL5* in combination with TET1CD. Interestingly, following dual expression of VP64 and TET1CD resulted in the fewest number of DE genes in RNAseq analysis. *In silico* analysis provided a predicted list of potential off-target genes either through base-pair mismatches or bulges in the gRNA. Only a single gene from the predicted off-target list, *CNTNAP2*, a gene implicated in autism-spectrum disorders (63), demonstrated differential expression following genome wide transcriptomics. Novel methodologies have been proposed to alter the binding specificities of sgRNAs in order to reduce off-target binding, such as engineering a hairpin secondary structure onto the sgRNA spacer region (64), and will be explored in future studies.

Up until recently, technical hurdles have hampered the assessment of the role of epigenetic heterogeneity in biological systems. One challenge that remains is whether the observed reactivation levels of *CDKL5* are due to a limited or partial reactivation at the population-wide level or if the observed effects are specific to a fully reactivated subgroup of cells. Recent evidence suggests that there are specific populations of cells that are more responsive to targeted effects, which will then drive the phenotype at the bulk level (28). It is possible that there are different kinds of responders to the epigenetic edits in our tested culture system and future studies will need to address this mechanistic question. Most likely this biological inquiry will need to be answered at a single cell level in future studies.

While this study has some limitations, reactivation strategies hold great promise for individuals suffering from X-linked disorders. In contrast to pharmacological inhibition of DNMT1, which postulates the need for mitosis, TET1CD might be a promising tool for demethylation in quiescent tissues that have been traditionally more difficult to target, such as the brain (27). In addition, superactivation by VP64 of the already active *CDKL5* allele needs to be carefully assessed due to the fact that Xp22 duplications containing the *CDKL5* gene have been described as pathogenic variants (65). Interestingly, we and others have identified that epigenetic editing using dCas9-TET1 does not exceed super-physiological levels of an X-linked target gene, further making this approach favorable in the light of a dosage sensitive gene. Future studies will have to assess what the biological consequence of the achieved escape level is on the gene and protein level in more disease-relevant models such as patient-derived iPSC-neurons, organoids, and animal models.

## DATA AVAILABILITY

UCSC Genome browser sessions

Fig. 1/sgRNA track:

https://genome.ucsc.edu/cgi-bin/hgTracks?db=hg19&lastVirtModeType=default&lastVirtModeExtraState=&virtModeType=default&virtMode=0&nonVirtPosition=&position=chrX%3A18442414%2D18444794&hgsid=760518777_CNw5jReTZafMY5caVcKMbQDyvAuJ

Fig. 3/bisulfite amplicon:

https://genome.ucsc.edu/cgi-bin/hgTracks?db=hg19&lastVirtModeType=default&lastVirtModeExtraState=&virtModeType=default&virtMode=0&nonVirtPosition=&position=chrX%3A18442414%2D18444794&hgsid=760518921_nzxZVSf7hLaEiNDs5A2a49axY4yT

Fig. 4/ChIP:

https://genome.ucsc.edu/cgi-bin/hgTracks?db=hg19&lastVirtModeType=default&lastVirtModeExtraState=&virtModeType=default&virtMode=0&nonVirtPosition=&position=chrX%3A18431699%2D18455508&hgsid=760518993_0RdFwo1mCQ1mil9pwGbhXa1jveKA

GEO series accession number GSE137668, token: alafkuqetnodbut

https://www.ncbi.nlm.nih.gov/geo/query/acc.cgi?acc=GSE137668

## Supplementary Material

gkz1214_Supplemental_Files
