# Supplementary material for: Artificial escape from XCI by DNA methylation editing of the CDKL5 gene
Source: Nucleic Acids Res. 2020 Jan 11;48(5):2372–87. doi: 10.1093/nar/gkz1214 (PMC7049732; doi:10.1093/nar/gkz1214)
Supplement: gkz1214_Supplemental_Files [file gkz1214_supplemental_files.zip › Supplementary_Figures__REVISED_.pdf]

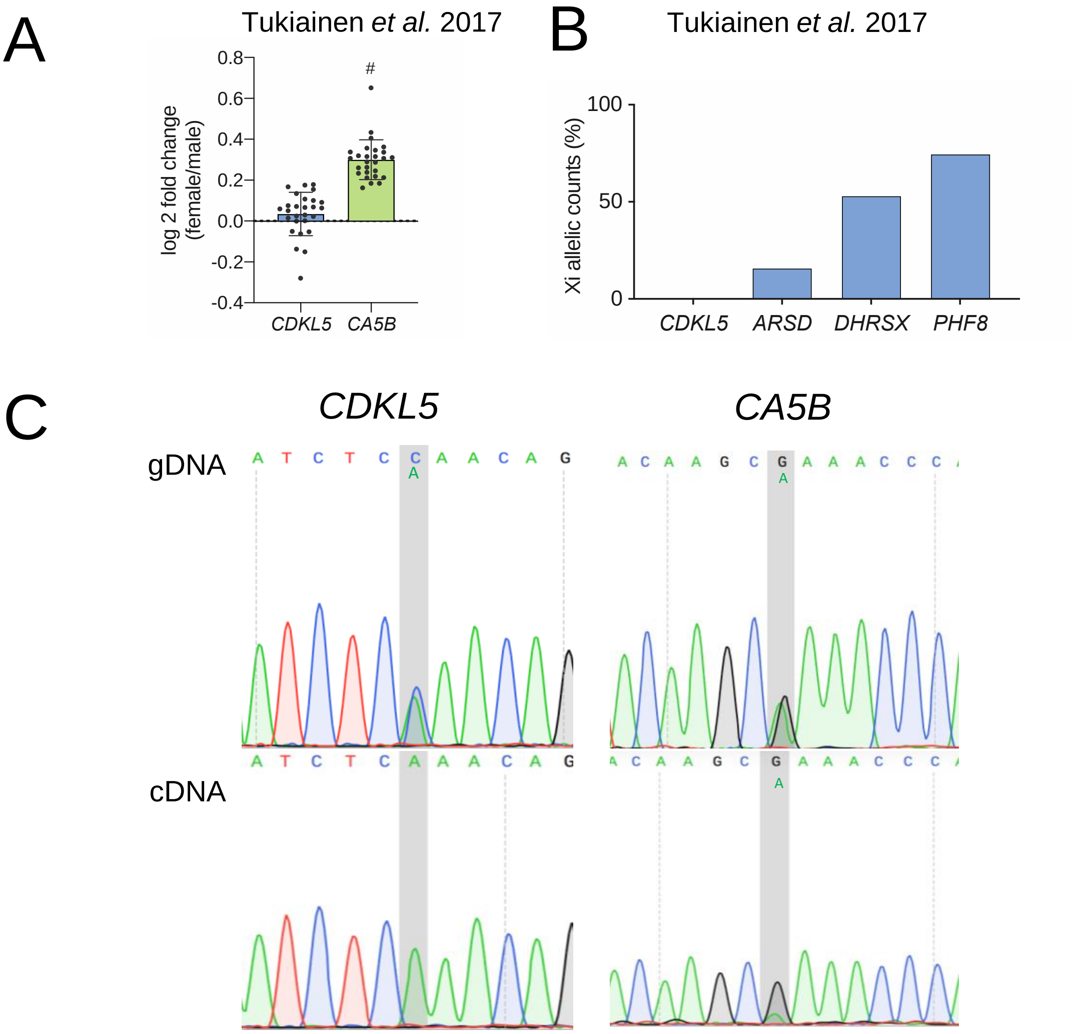

Supplementary Figure 1. (**A**) Male–female expression differences in *CDKL5* and a known XCI escape gene *CA5B* across 27 GTEx tissues. (**B**) Analysis of XCI status of *CDKL5* and genes showing variable expression from the inactive X-chromosome using scRNA-seq from previously published data (16). #Significantly different from *CA5B*, $p < 0.05$. (**C**) Sanger sequencing of genomic DNA and cDNA from SH-SY5Y to confirm that *CDKL5* shows mono-allelic expression of a SNP, in contrast to an escape gene, *CA5B*, which shows expression from the escape allele.

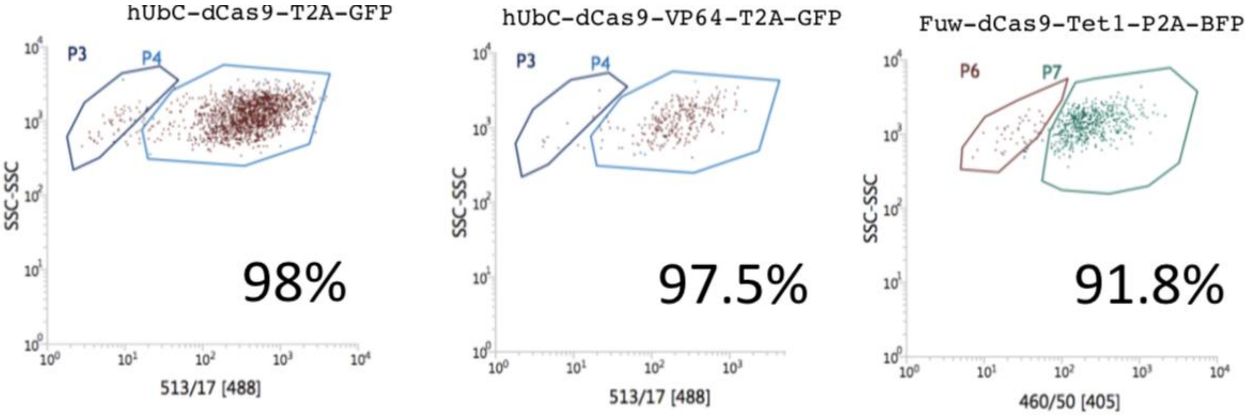

Supplementary Figure 2. Flow analysis of cells purified post-enrichment by FACS to stably express dCas9 or dCas9-VP64 fused to a GFP via a a T2A peptide or dCas9-TET1CD-P2A-BFP.

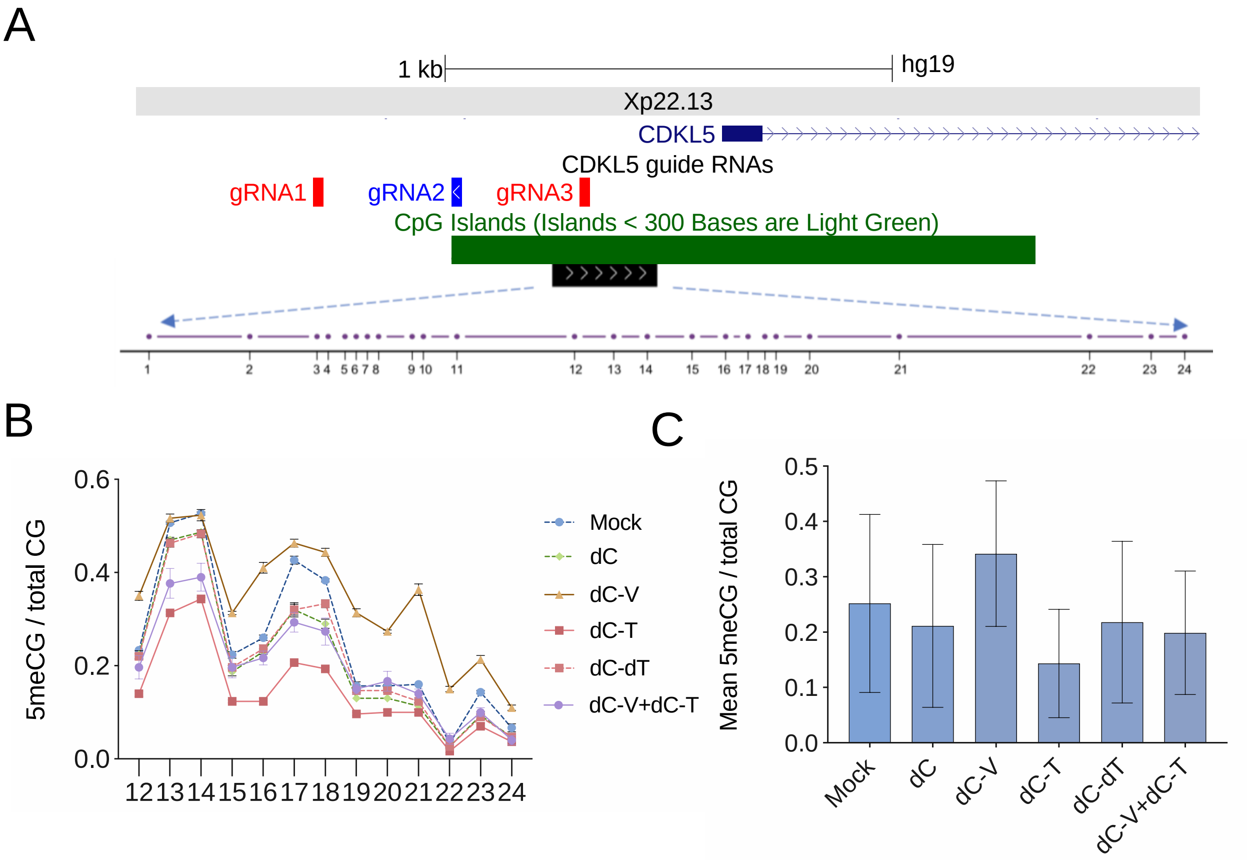

Supplementary Figure 3. dCas9-TET1CD does not remove DNA methylation distal to the *CDKL5* CGI promoter target site. (**A**) UCSC genome browser snapshot of the target sites of sgRNAs 1-3 directed against the *CDKL5* promoter on Xp22.13 and a large CpG Island (>1kb) spanning the transcriptional start site of *CDKL5*. The black box represents a >200 bp region assessed for targeted DNA methylation changes containing 24 individual CpG dinucleotides (drawn to scale). (**B**) 5-methylcytosine levels in a CpG context (5meCG) over total CpG context as assessed by targeted bisulfite sequencing across CpG dinucleotides 12 - 24 in mock-treated cells or cells transduced to constitutively express dCas9-no effector (dC) or dCas9 fused to either VP64 (dC-V) or TET1CD (dC-T), a combination thereof (dC-V+dC-T) or a catalytically inactive TET1CD (dC-dT). X-axis depicts the individual CpG position relative to the amplicon (not drawn to scale). (**C**) Mean 5-methylcytosine levels in a CpG context over all 12 CpG dinucleotides in all treatment groups, *n* = 3 independent experiments.

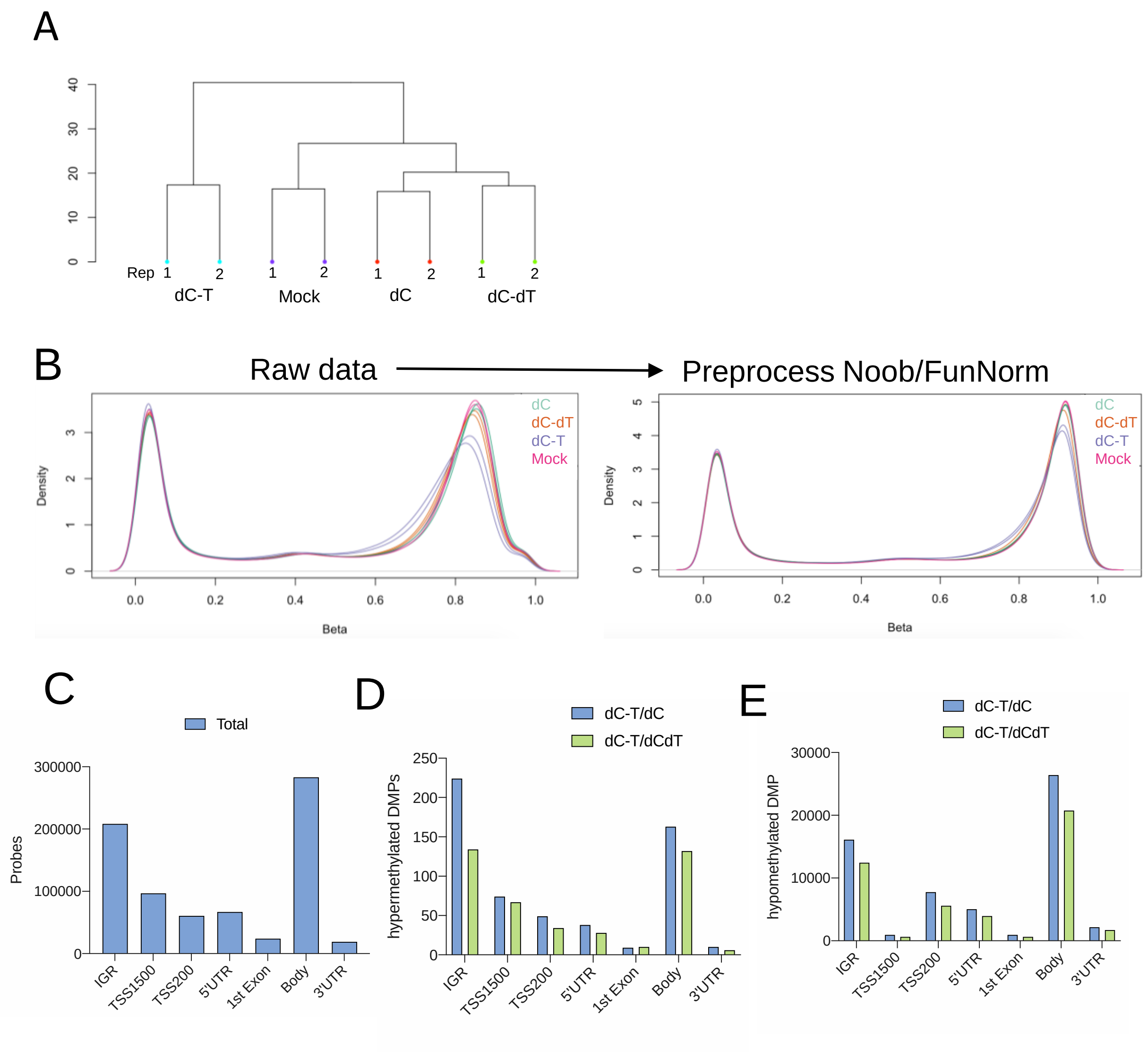

Supplementary Figure 4. QC analysis of 850K MethylationEPIC data. (**A**) A dendogram showing that biological replicates cluster together and that controls show different hierarchies then dCas9-TET1CD. (**B**) Density plots of beta value distribution before and after normalization with preprocessNoob and preprocessFunNorm. (**C**) Total probe statistics by feature. (**D**) Total number of hypermethylated differentially methylated positions by feature. **(E**) Total number of hypomethylated differentially methylated positions by feature.

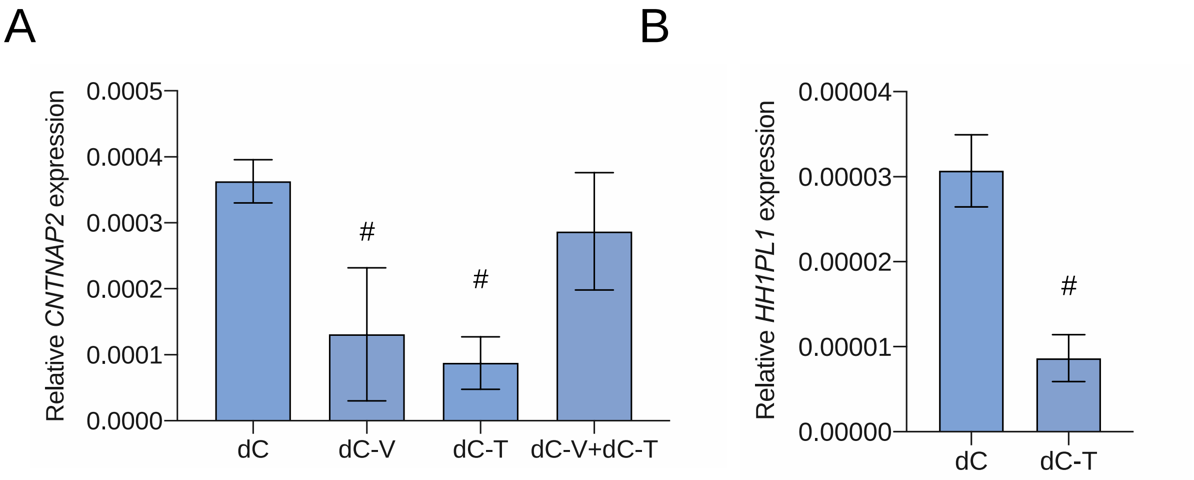

Supplementary Figure 5. Validation of differentially expressed genes by RT-qPCR. (A) Relative *CNTNAP2* mRNA in SH-SY5Y determined by RT-qPCR after constitutive expression of dCas9 (dC), dCas9-VP64 (dC-V), dCas9-TET1CD (dC-T) or a combination of dCas9-VP64 and dCas9-TET1CD (dC-V+dC-T) and sgRNAs 1-3 after 21 days post-transduction. #Significantly different from dCas9, n = 3 independent experiments, Tukey's HSD, $p < 0.05$. #Significantly different from dCas9 sgRNAs 1-3, n = 3 independent experiments, Student t-test $p < 0.05$. (B) Relative *HHIPL1* mRNA in SH-SY5Y determined by RT-qPCR after constitutive expression of dCas9 (dC) or dCas9-TET1CD (dC-T) and sgRNAs 1-3 after 21 days post-transduction. #Significantly different from dCas9 sgRNAs 1-3, n = 3 independent experiments, Student's t-test, $p < 0.05$.
